# Maize monoculture supported pre-Columbian urbanism in southwestern Amazonia

**DOI:** 10.1038/s41586-024-08473-y

**Published:** 2025-01-29

**Authors:** Umberto Lombardo, Lautaro Hilbert, McKenzie Bentley, Christopher Bronk Ramsey, Kate Dudgeon, Albert Gaitan-Roca, José Iriarte, Andrés G. Mejía Ramón, Sergio Quezada, Marco Raczka, Jennifer G. Watling, Eduardo Neves, Francis Mayle

**Affiliations:** 1 Institute of Environmental Science and Technology, Universitat Autònoma de Barcelona, Barcelona, Spain; 2 Department of Prehistory, Universitat Autònoma de Barcelona, Barcelona, Spain; 3 Museum of Archaeology and Ethnology, University of São Paulo, São Paulo, Brazil; 4 School of Archaeology, University of Oxford, Oxford, UK; 5 School of Ocean and Earth Science, University of Southampton, Southampton, UK; 6 Department of Archaeology and History, University of Exeter, Exeter, UK; 7 Trinidad, Bolivia; 8 School of Geography, University of Nottingham, Nottingham, UK; 9 Department of Geography and Environmental Science, University of Reading, Reading, UK

**Keywords:** Archaeology, Ecology

## Abstract

The Casarabe culture (500–1400 ce), spreading over roughly 4,500 km^2^ of the monumental mounds region of the Llanos de Moxos, Bolivia, is one of the clearest examples of urbanism in pre-Columbian (pre-1492 ce) Amazonia. It exhibits a four-tier hierarchical settlement pattern, with hundreds of monumental mounds interconnected by canals and causeways^1,2^. Despite archaeological evidence indicating that maize was cultivated by this society^3^, it is unknown whether it was the staple crop and which type of agricultural farming system was used to support this urban-scale society. Here, we address this issue by integration of remote sensing, field survey and microbotanical analyses, which shows that the Casarabe culture invested heavily in landscape engineering, constructing a complex system of drainage canals (to drain excess water during the rainy season) and newly documented savannah farm ponds (to retain water in the dry season). Phytolith analyses of 178 samples from 18 soil profiles in drained fields, farm ponds and forested settings record the singular and ubiquitous presence of maize (*Zea mays*) in pre-Columbian fields and farm ponds, and an absence of evidence for agricultural practices in the forest. Collectively, our findings show how the Casarabe culture managed the savannah landscape for intensive year-round maize monoculture that probably sustained its relatively large population. Our results have implications for how we conceive agricultural systems in Amazonia, and show an example of a Neolithic-like, grain-based agrarian economy in the Amazon.

## Abstract (web summary)

Remote sensing, field survey and microbotanical analyses show that the pre-Columbian Casarabe culture in the Bolivian Amazon invested heavily in landscape engineering, constructing a complex system of drainage canals and newly documented savannah farm ponds.

## Main

The role of grain agriculture as the subsistence base of prehistoric complex societies in both the Old and New World has been a matter of sustained debate for many decades (see, for example, refs. ^4–8^). In Mesoamerica, the earliest evidence of maize as a staple crop dates to 4,000 calendar years before the present^9^. The timing and nature of maize’s role as the staple crop of Andean civilizations, as seen in early historical accounts, is controversial (see, for example, refs. ^6,10^). In Amazonia it is well established, from both archaeological and palaeoecological data, that maize has been cultivated since at least 6,850 calendar years before the present^11^; however, to date there is no evidence of it being a staple crop. Most societies had mixed economies relying on multiple cultigens^12–16^. Roosevelt^17^ proposes that the rise of social complexity in the Amazon was based on maize agriculture. However, current archaeological evidence has not been conclusive of maize cultivation being the staple crop of complex societies of the Amazon^15^. Current archaeobotanical and palaeoecological data from Late Holocene complex societies in Amazonia indicate polyculture (mixed-cropping) agroforestry, not maize monoculture, as the basis of a subsistence economy^15,18–22^.

Recent archaeological research has revealed evidence for low-density urbanism, social complexity and large populations in the Andean foothills of the Upano River region of Ecuador^23^, and in the monumental mounds region (MMR) in the seasonally flooded savannahs of the Bolivian Amazon^1^. Here in the MMR, the Casarabe people built hundreds of monumental mounds interconnected by canals and causeways across a flat forest–savannah mosaic landscape dominated by seasonally flooded savannahs, with forests restricted to non-flooded palaeo-river levées. Whereas drained fields and terraces, built on extremely fertile volcanic soils, were clearly integral to low-density agrarian urbanism of the Upano region^23^, the type of farming system needed to sustain the Casarabe culture is still unknown. It has been proposed that the construction of drainage canals permitted cultivation of the relatively fertile sediments of the seasonally flooded savannahs of the MMR^24^ without the need for deforestation^25^. However, no agricultural fields or other food production systems have hitherto been found in connection with such canals, leaving unanswered the question of how the Casarabe people managed to feed its relatively large population. To address this issue, we combine remote-sensing imagery with a programme of coring, test pits, radiocarbon dating and pollen and phytolith analyses on both seasonally flooded savannahs and forest.

**Fig. 1:**
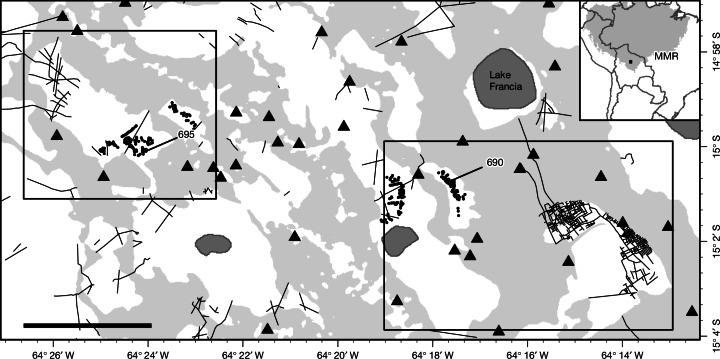
Two examples of engineered landscape in the MMR. Triangles represent monumental mounds; black dots, ponds; thin black lines, canals; light grey areas, forest; white areas, savannah; and dark grey areas, lakes. Inset top left, the northern side of a savannah is crisscrossed by canals but the southern side is dotted with ponds. Inset bottom right, one savannah (to the east) has been modified by the excavation of a densely packed network of drainage canals, and two other savannahs, to the west, are dotted with ponds; in the former, the network of canals drains into Lake Francia located about 4 km to the north. 690 and 695 indicate the locations of the two farm ponds sampled for this study. Inset top right, boundaries of Amazonia as defined in ref. ^43^ and the image of World Countries Generalized provided by ESRI under the ArcGIS Pro licence. Scale bar, 5 km. Credit: European Commission JRC.

**Extended Data Fig. 1:**
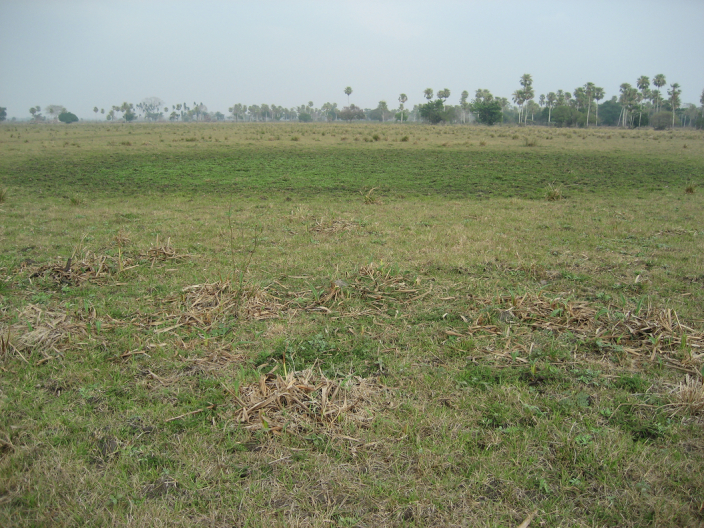
Field view of a circular farm pond. The diameter of the pond is ca. 70 metres.

We have identified two unreported and complementary agrotechnologies in the savannahs of the MMR: dense drainage networks and artificial farm ponds (Fig. 1 and Extended Data Fig. 1), in which different portions of a savannah (Fig. 1, top left inset), or different savannahs within the same area (Fig. 1, bottom right inset), have been heavily modified—into either intricate arrangements of canals or clusters of circular depressions.

## The drainage network

**Fig. 2:**
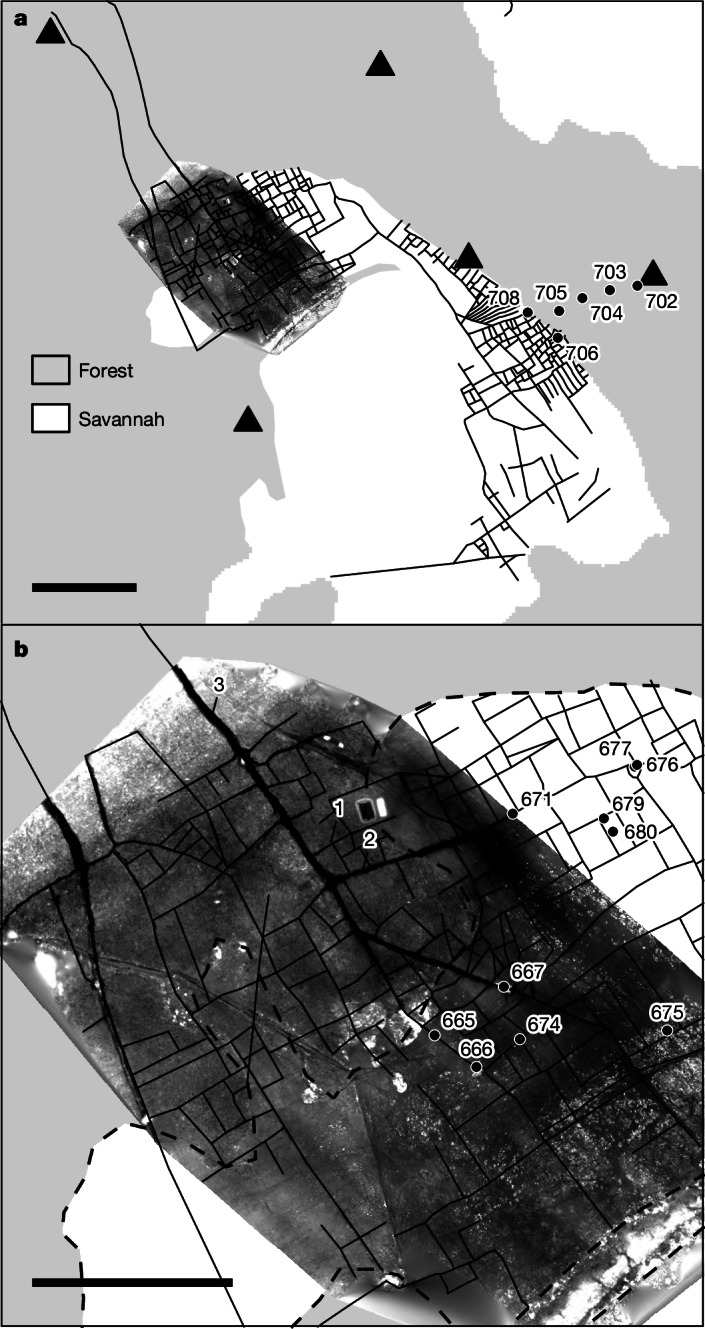
Drainage canals and digital elevation model. **a**, The complete drainage network. Numbered dots indicate the location of phytolith profiles. **b**, Detail of the digital elevation model of the northern part of the drainage network. Dashed lines represent the forest–savannah boundary, showing that a great deal of the drainage network is presently covered by forest. See Fig. 1b for location. Scale bars, 1 km (**a**), 500m (**b**).

**Extended Data Fig. 2:**
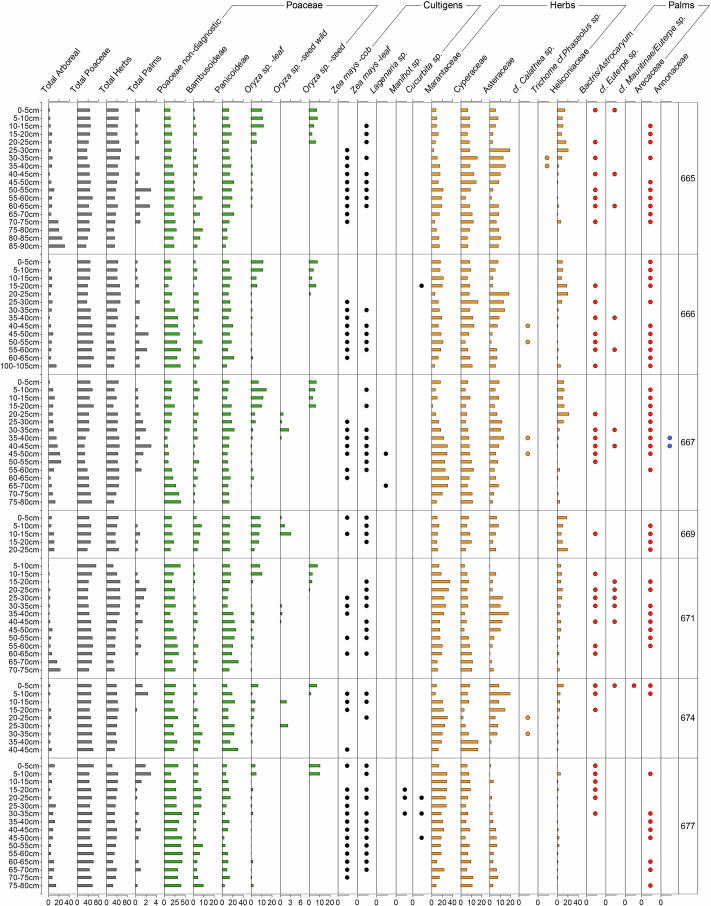
Phytolith profiles of drainage canals. Horizontal bars represent percentages; circles correspond to presence of plant taxa lower than 1% in abundance. See Fig. 2 of the main text for location.

In one of the savannahs under study (Fig. 2), the small canals converge into larger canals that drain the whole savannah toward Lake Francia to the north (Fig. 1b). We identified three orders of drainage canals: the first order (1 in Fig. 2b), the smallest, are around 4 m wide and 25 cm deep, the second order (2 in Fig. 2b) are around 8 m wide and 70 cm deep and the main canal (third order) that drains into the lake is 14 m wide and 1.8 m deep (3 in Fig. 2b), becoming 3.2 m deep about 1.5 km before reaching the lake. Overall, the drainage network drains towards the north, becoming ever deeper with respect to the general topography. Several stratigraphic profiles of the canals show that the original depth of the canal network was around 80 cm deeper than at present for the second-order canals (see profiles 667 and 671 in Extended Data Fig. 2) and around 45 cm deeper for the first-order canals (for example, profile 674 in Extended Data Fig. 2). The drainage network is associated with circular elevated platforms roughly 50 cm in height, resembling pre-Columbian forest islands^11^, and with small mounds of around 2–3 m in diameter. The elevated platforms are surrounded by deep canals (profiles 666 and 677 in Extended Data Fig. 2).

Soil cores were collected from several locations both inside the canals and between them (Fig. 2b). Phytolith analysis shows a high abundance of phytoliths derived from the cob glumes and leaves of *Zea mays* in almost all canal soil profiles (Extended Data Fig. 2), with sporadic presence of *Cucurbita* spp. (666 and 677), *Manihot* sp. (677)*, Calathea* sp. (674) and *Lagenaria* sp. (667) phytoliths. We cannot exclude the possibility that *Cucurbita* was cultivated in greater amounts than implied by the phytolith assemblage, because some domesticated *Cucurbita* varieties may lack scalloped phytoliths^26^.

**Extended Data Fig. 3:**
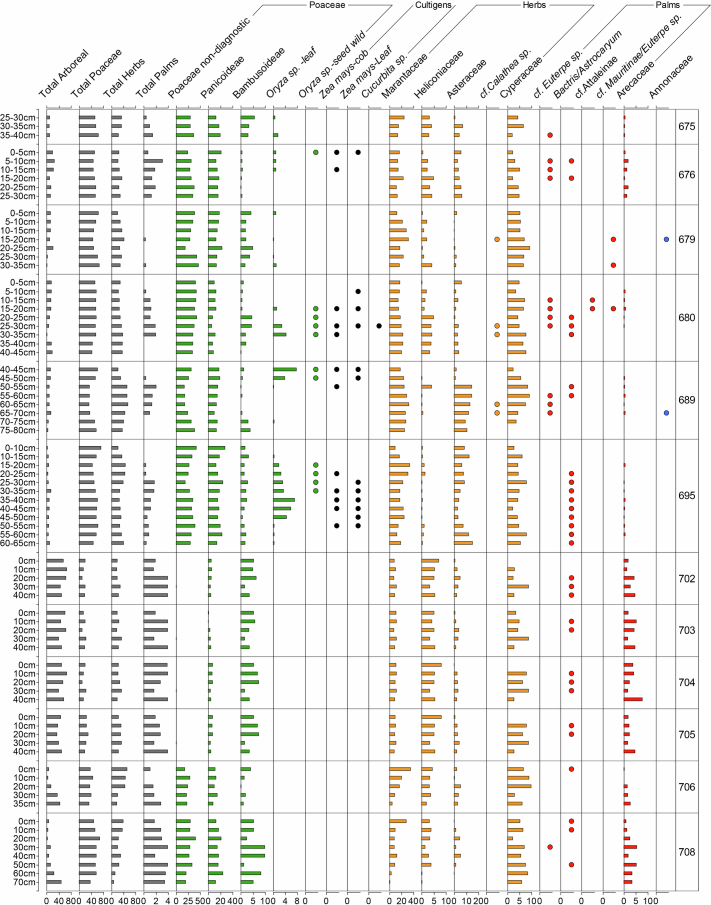
Phytolith profiles of causeway (675), fields (676, 679,680, 706), ponds (689, 695), forest (702, 703, 704, 705) and canal (708). Horizontal bars represent percentages; circles correspond to presence of plant taxa lower than 1% in abundance. See Fig. 2 of the main text for location.

**Extended Data Fig. 4:**
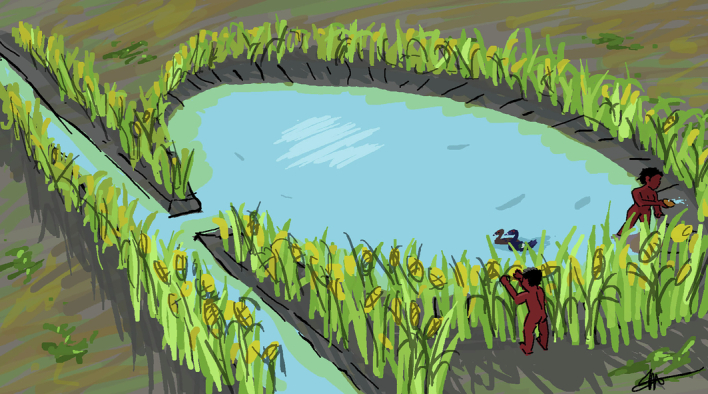
Artwork representing how farm ponds and drainage canals were probably used for maize agriculture. Maize was planted around the pond and along the edges of the canals. Canals and ponds are here depicted together for simplicity. Credit: J. P. Guevara.

The majority of *Oryza* phytoliths are concentrated in the upper levels of all the profiles. Most upper-level glume phytoliths were from domesticated plants (Methods), whereas those from lower levels in the soil profiles (30–50 cm depth) were classified as wild species. These results are not surprising, because some of these fields are currently being used to grow modern Asian rice^27^. The low production of diagnostic wavy-top rondel phytoliths in maize^28^ and the high abundance of these, relative to the sporadic presence of phytoliths of other cultivars, indicate that maize was by far the principal cultivar in these savannahs. We attribute the absence of maize phytoliths in the uppermost 20–25 cm of the canal soil profiles to sedimentary fill from adjacent fields over recent decades or centuries—an inference corroborated by our soil phytolith data from fields between the canals (Extended Data Fig. 3). Here, maize phytoliths appear in only one of the three profiles, suggesting that the cultivated area is likely to have been established along the canals, probably on elevated rims that have since eroded into the canals. It is probable that, while in use, the original depth of the canals was maintained by their periodic re-excavation and redistribution of canal sediment fill along the canal margins, where maize was then planted (Extended Data Fig. 4), mimicking what has been proposed for raised-field agriculture in other regions of the Llanos de Moxos^29^.

## The forest

Forest in the study region grows on elevated surfaces, mostly fluvial levees, that remain above the water level during the rainy season. Four soil profiles were dug and sampled across the forest, along a transect from the savannah to a large 15-ha monumental mound (Fig. 2a and Extended Data Fig. 3), to reveal to what extent the forest was cleared for agriculture. No charcoal or any other evidence of fire was visible in any of the profiles. Phytolith profiles are all similar and do not show any obvious stratigraphic change, apart from a slight reduction in Arecaceae (palms) and an increase in Poaceae (grass) phytoliths, which could indicate a slight opening of the forest canopy. No cultivar phytoliths were found in any of the profiles (Extended Data Fig. 3). Although our data cannot show the extent to which the forest was used for agroforestry, wood harvesting, hunting or cultivation of medicinal plants, the absence of charcoal does show that slash-and-burn agriculture did not take place here.

## The clusters of farm ponds

**Fig. 3:**
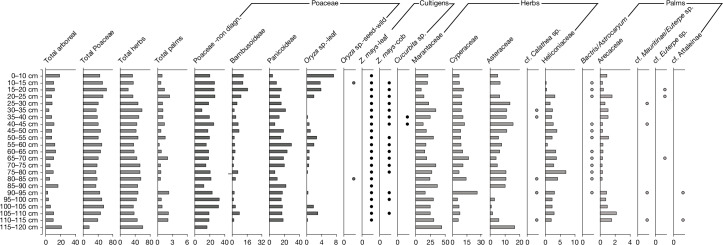
Relative frequencies of phytoliths recovered from farm pond, profile 690. Horizontal bars represent percentages, circles correspond to the presence of plant taxa lower than 1% in abundance. Vertical axis is the depth of the sample in cm. Source data are provided in Supplementary Data 1. non-diagn., non-diagnostic.

**Extended Data Fig. 5:**
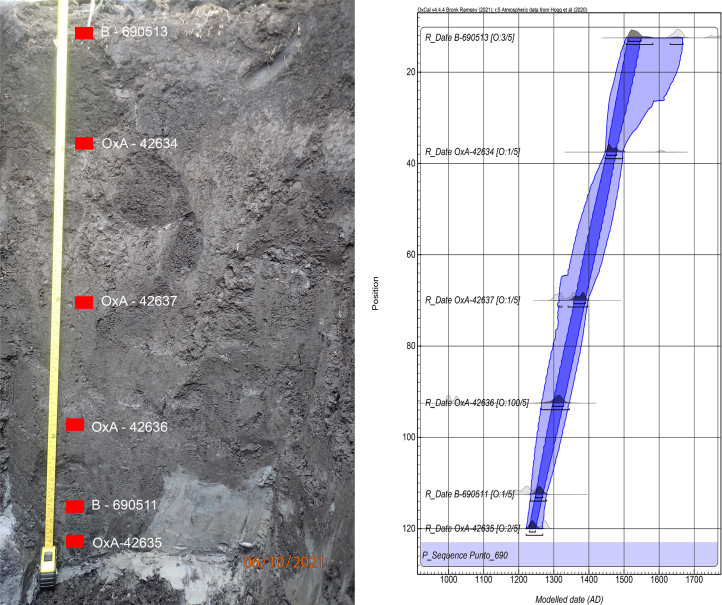
Profile of pond 690 with associated age/depth model. The pond was in use for ca. 300 years, from ca. 1250 to ca. 1550 Cal yrs. ce. Samples for radiocarbon ages have been collected along the yellow measuring tape shown in the figure.

**Extended Data Fig. 6:**
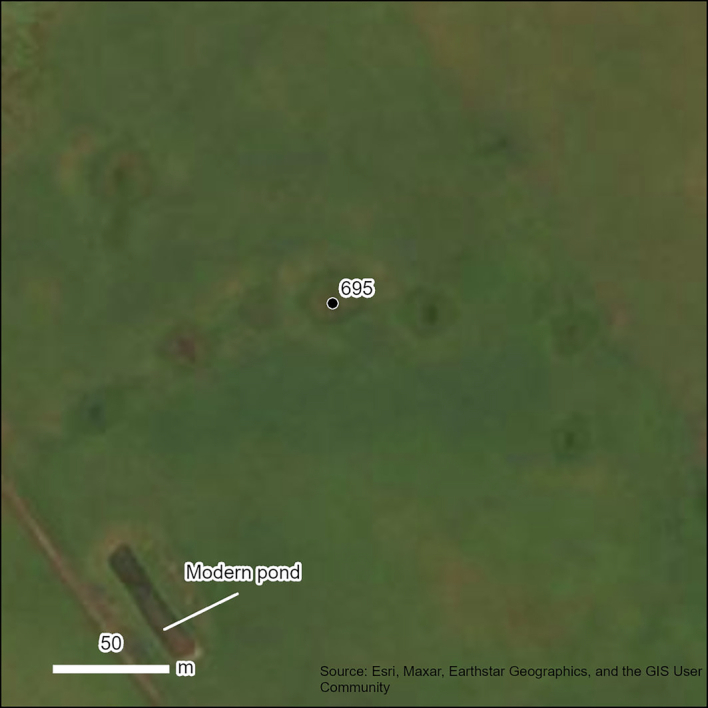
Pond cluster with location of profile 695. Most of the ponds are aligned and are connected by depressions/canals. Image provided by ESRI under the ArcGIS Pro license.

**Extended Data Fig. 7:**
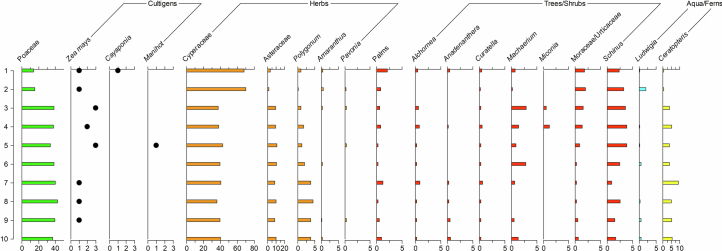
Diagram of relative frequencies of pollen recovered from farm pond, profile 690. Horizontal bars represent percentages; circles correspond to the absolute number of pollen grains. Source data are provided in Supplementary file Pollen.xls.

**Extended Data Fig. 8:**
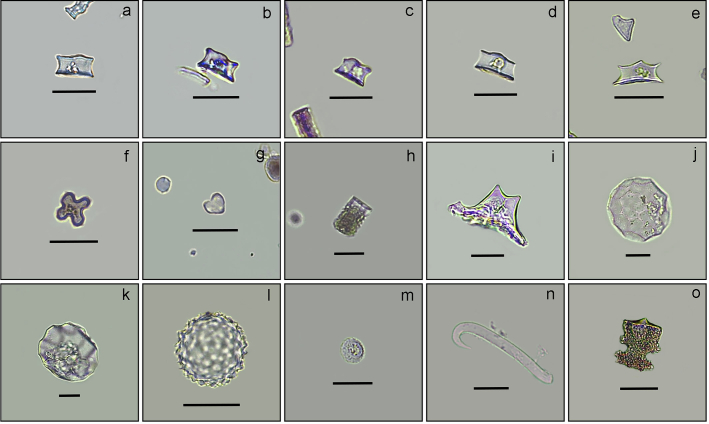
Microphotographs showing examples of key phytolith morphologies identified and their taxonomic and anatomical associations. (A-D) Zea mays cob, WAVY-TOP RONDEL (A-690 80-85 cm; B-690 40-45 cm; C-690 65-70 cm; D-666 65-65 cm); (E) Poaceae non-diagnostic to maize leaf/stem/inflorescence, spiney WAVY-TOP RONDEL (666 40-45 cm); (F) Poaceae leaf, CROSS VARIANT-1 (695 30-35 cm); (G) Manihot sp. secretory cell, HEART-SHAPED (677 20-25 cm); (H) cf. Calathea sp. rhizome, FLAT DOMED CYLINDER (690 30-35 cm); (I) Oryza sp. husk, DOUBLE-PEAK GLUME (695 20-25 cm); (J) Cucurbita sp. rind, SCALLOPED SPHEROID (680 25-30 cm); (K) Lagenaria sp. rind, irregularly-shaped SCALLOPED SPHEROID (667 45-50 cm); (L) cf. Euterpe sp. all plant parts, large dense SPHEROID ECHINATE (667 30-35 cm); (M) Bactris/Astrocaryum all plant parts, CONICAL TO HAT-SHAPED BODY (665 20-25); (N) Multiple plants species (e.g. Commelinaceae and Phaseolus sp.) trichome, HOOK-SHAPED HAIR (665 30-35 cm); (O) Marantaceae seed, CILINDRICAL BODY (671 35-40 cm). Scale = 20 µm.

A large portion of the savannahs in the MMR contains clusters of circular depressions of 10–100 m in diameter. They are often connected, either by canals or directly by adjacency. These ponds are similar to natural depressions called *gilgais*, an Aboriginal Australian name for water holes, that form in vertisols because of repeated expansion and contractions of the clay^30^. To understand their genesis and use, we sampled two ponds in two different savannahs. Profiles 690 (Fig. 3) and 689 (Extended Data Fig. 3) were excavated and cored, respectively, in a large pond of roughly 100 m in diameter, with its central depression about 60 cm below the surroundings. Profile 695 (Extended Data Fig. 3) was excavated in a pond of roughly 30 m in diameter, and with a central depression currently 40 cm below the surrounding savannah. Pond profile 690 exhibits a very irregular, sharp contact between the organic sediment fill and the grey, inorganic clay below (Extended Data Fig. 5), and shows no evidence of shear surfaces (slickensides), suggesting that the pond was excavated and is not a *gilgai*. The anthropogenic origin of these depressions is further supported by their size, which is far larger than the 15–20-m-diameter *gilgais*^31,32^, and by their clustered linear distribution (Extended Data Fig. 6). Sediment profiles from both ponds show the continuous presence of maize phytoliths and pollen (Fig. 3 and Extended Data Fig. 7) throughout, with phytoliths of *Cucurbita* sp. present in only two adjacent samples in profile 690 at around 40 cm depth, and a pollen grain of Manihot at approximately 50 cm depth (Extended Data Figs. 3, 7 and 8). No other cultivars were detected. The chronology of pond profile 690 indicates that this system was in use around 1250–1550 calendar years ce (Extended Data Fig. 5).

**Extended Data Fig. 9:**
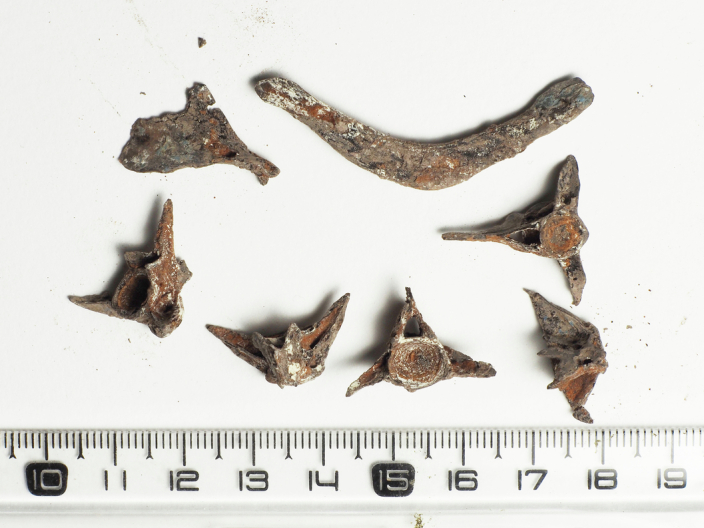
Bones of *Synbranchus marmoratus* found in pond 695 at a depth of 70 cm. Bones of *S. marmoratus* are often found in monumental mounds. It hibernates in the mud and it is a predictable and ready available source of proteins. Scale bar in cm.

Today, the majority of these ponds hold water for most of the year, maintaining wet soil until the very end of the dry season. If this hydrological balance is representative of the past, these ponds would have provided sufficient water for maize cultivation around their margins throughout the dry season. This is not dissimilar to the *k’hochas* in the Bolivian altiplano^33^, where yields are up to four times higher than for regional rainfed production. Similar pond-based farming systems have been described in Bangladesh and India^34,35^, where ponds provide an integrated production system that includes fish farming, poultry and cultivation of pond dykes. A very similar system could have been in place in the MMR, where swamp eels (*Synbranchus marmoratus*) were an important part of the diet of the Casarabe culture^36^; the Muscovy duck (*Cairina moschata*), the only known domesticated animal in the Amazon^37^, was probably kept^38,39^. Bones of *S. marmoratus* were found in pond 695 at a depth of 70 cm (Extended Data Fig. 9). During the dry season, these farm ponds would have served as ‘watering holes’, attracting game.

## A pre-Columbian green revolution

The combination of these two types of landscape engineering—drainage canals and farm ponds—is unique to the MMR. We argue that it was a highly innovative agricultural strategy that enabled the Casarabe culture to substantially increase the cultivation period for maize, as well as providing easy access to fish, birds and game. Through the sophisticated system of drainage canals, some savannah wetlands were converted into drained fields suitable for intensive maize monoculture in the wet season, whereas the construction of clusters of farm ponds in other savannahs provided a reservoir of water that allowed pot irrigation, which enabled the continuation of maize agriculture throughout the dry season. The combination of these two water management systems would have allowed at least two harvests of maize per year. The lack of any evidence of cultivation and fire in the nearby forested areas suggests that slash-and-burn agriculture was unlikely to have been practised. Instead, this pre-Columbian Casarabe culture probably preserved the spatially limited, and hence highly valuable, forest resource for other key ecosystem services, such as firewood, building materials, medicinal plants and probably polyculture agroforestry. These data are corroborated by palaeoecological studies in the MMR that show no substantial change in forest cover^25^ during the Casarabe culture period. There are at least seven monumental mounds surrounding the drainage network and five surrounding the savannah with the pond cluster, which includes profile 690. These form part of the four-tier settlement pattern belonging to a political structure that guaranteed food production and agricultural infrastructure maintenance for hundreds of years^1,2^. Even though micro- and macrobotanical remains from monumental mounds show the presence of a variety of food and industrial crops, including maize, manioc, lerén, squash, peanuts, cotton, yams and palms^40,41^, our data suggest that maize was the staple crop for the Casarabe culture when the drainage and pond agricultural system was in operation. Our data show that the absence of other cultigen pollen in MMR lake cores^25^, and the greater abundance of maize macroremains, phytoliths and starch grains in the sediments and ceramics of Mendoza and Salvatierra monumental mounds^40,41^, is not due to low pollen productivity/preservation or taphonomic bias, but instead reflects a real phenomenon of greater reliance on maize in the diet compared with other cultigens.

Our results overturn the assumption that the seasonally flooded savannah of southwestern Amazonia is suitable only for cattle ranching and intensive Asian rice agriculture, and unsuitable for nutrient-demanding crops such as maize. These findings have implications for our understanding of pre-Columbian subsistence economies across Amazonia and beyond. They indicate that, during the late Holocene, alongside intensive polyculture (mixed-cropping) agroforestry on Amazonian Dark Earths^15^, other agricultural systems such as drained fields and farm ponds in the Llanos de Moxos were primarily focused on the cultivation of maize. These practices bear similarities to agricultural strategies observed in later Andean states and chiefdoms^42^. Collectively, as long argued, intensive cultivation of maize has had a major role in supporting the economy of some of the most complex societies in the Americas. The Casarabe people demonstrated the ability to establish a highly intensive monoculture farming system on the savannahs based on maize, maintaining the surrounding forest cover and supporting one of the most complex pre-Columbian societies in lowland South America. The Casarabe culture of the MMR provides a clear example of when the rise of social complexity is linked to intensive food production and, more specifically, to maize monoculture. It also confirms the role of grain agriculture as the main driver for increasing social complexity and, probably, inequality^7^.

## Methods

### Phytolith processing and identification

Phytoliths were extracted from sediments following previously published methods^44^. Phytoliths were identified and counted using a Zeiss Axioscope 40 light microscope at ×500 magnification. Phytolith identifications were made using published material for the Neotropics^45–49^, and by direct comparison with the phytolith reference collection of the Archaeobotany and Palaeoecology Laboratory (Department of Archaeology, University of Exeter, UK) and at the phytolith laboratory of ICTA-UAB (Universitat Autónoma de Barcelona). A minimum of 200 diagnostic randomly placed phytoliths were counted per slide. A full scan of slides was performed to detect the presence of squash, manioc and maize. The average size of attributes measured on *Oryza* glume phytoliths identified in the pond/canal systems followed the model proposed by Hilbert et al.^50^. Overall, all glume phytolith mean width and height measurements were compared with the Monte Castelo site to assess the likelihood of a domesticated origin. Glume phytoliths identified in upper layers from sites at which *O. sativa* is currently cultivated were analysed using prediction calculations proposed by Zhao et al.^51^. We confirmed the presence of Asian domesticated rice on all upper layers. Overall, our analysis indicates that the origin of rice phytoliths from our samples was statistically similar to both wild botanical specimens (*Oryza latifolia* and *Oryza alta*) and lower layers (I–J) from the Monte Castelo site^50^.

### Pollen processing and identification

Samples for pollen analysis were treated following a protocol designed to improve the recovery of large pollen grains—in particular, those of cultigens^52^. Two tablets of the exotic marker *Lycopodium clavatum* were added to each sample to facilitate the calculation of pollen concentration per cubic centimetre^53^. Pollen and spores were analysed using a Leica DMLB microscope at ×400 and ×1,000 magnification, and identifications were made using the modern pollen reference collection at the University of Reading, as well as the Neotropical pollen database^54^ and specialized atlases^55–57^. In every sample, a total of 300 randomly placed terrestrial pollen grains were counted.

### Drone light detection and ranging

A light detection and ranging survey was conducted using a Zenmause L1 sensor mounted on a Matrice 300 real-time kinematic (RTK) drone and a D-RTK 2 base station. We used a postprocessing kinematic solution rather than RTK for data correction, because of malfunctioning of the latter device. Four flights at an altitude of 100 m and speed of 6 m s^−1^ were needed to cover the entire area; point density was 477 m^−2^. The missions were planned with DJI Pilot 2, v.9.0.5.5. We set the sensor to detect three returns, its maximum limit, to ensure the recording of laser bounce on the ground through the tree canopy, which covered around 50% of the surveyed area. Data were processed using D-RTK 2 data in the postprocessing kinematic workflow of DJITerra software according to the Zenmuse L1 v.1.1 operation guidebook^58^. Terramatch software v.023.014 was used in the Spatix environment to align datasets, correct trajectories, delete overlapping points and smooth noise points, following the workflow steps explained in the user guide.

### Radiocarbon dating

Accelerator mass spectrometry radiocarbon dating was performed on seven samples from profile 690 at the Oxford Radiocarbon Accelerator Unit and Beta Analytic; dates are reported in Extended Data Table 1. The samples dated at the Oxford Radiocarbon Accelerator Unit were chemically pretreated using an acid–base–acid protocol for the insoluble humin fraction of sediments, and subsequently dated following their protocols^59^. The same acid–base–acid protocol was used by Beta Analytic. Radiocarbon dates were calibrated using SHCAL20 (ref. ^60^), modelled using the P_Sequence command and outlier modelling in OxCal v.4.4.4 (refs. ^61–64^). The code used is available in Supplementary Information.

### Inclusion and ethics

The study included several South American researchers (S.Q., J.I., L.H., E.N. and M.R.) who contributed to various aspects of the research project. The research is locally relevant, and several local institutions (Gobernación del Beni, Universidad Autonoma del Beni and Alcaldía de Trinidad) have repeatedly expressed public support. We have a collaboration Agreement with CIBIOMA at Universidad Autónoma del Beni José Ballivián for training of local students in phytolith analysis (we are currently setting up a laboratory in Trinidad). We have provided training and materials to Museo Etnoarqueológico Kenneth Lee in Trinidad. The type of study we performed did not require the approval of a local ethics review committee. The local and regional research relevant to our study has been taken into account in citations.

### Reporting summary

Further information on research design is available in the Nature Portfolio Reporting Summary linked to this article.

## Online content

Any methods, additional references, Nature Portfolio reporting summaries, source data, extended data, supplementary information, acknowledgements, peer review information; details of author contributions and competing interests; and statements of data and code availability are available at 10.1038/s41586-024-08473-y.

## Supplementary information

Supplementary Data 1 Raw phytolith data. This file contains the phytoliths for all samples analysed in the study, including the raw data used for Fig. 3 and Extended Data Figs. 2 and 3. In the tab ‘%’, count is expressed as a percentage; in tab ‘number’, the absolute number of phytoliths was counted for each sample.

Reporting Summary

Supplementary Data 2 Raw pollen data. This file contains the data used for Extended Data Fig. 7. In tab ‘PollenRawData’, the absolute number of pollen grains was counted for each sample; in the tab ‘PollenPercentageData’, the count is expressed as a percentage.

## Figures and Tables

**Extended Data Table 1:**
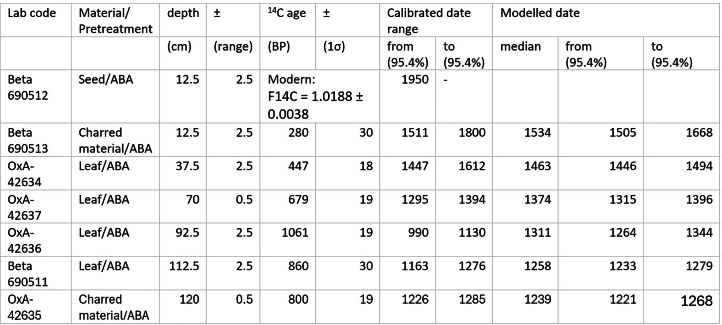
Radiocarbon dates conducted on pond 690 and used in the associated Bayesian age depth model

The date ranges are given as highest probability density ranges at the 95.4% level;the modelled dates are taken from Model 1 referred to in supplementary information which is an age-depth model and so the dates are estimates for the dates of the sediment at the given depths rather than estimates of age of the samples where they are outliers; for the modelled dates the median date for the posterior distributions is also given. ABA stands for acid-base-acid.

## Data Availability

All phytolith and pollen data supporting the findings of this study are available in Supplementary Information. Phytoliths were identified using the sources referenced in Methods. Pollen was identified using the Neotropical pollen database (https://research.fit.edu/paleolab/pollen-database/) and the sources referenced in Methods.
