# Supplementary material for: Maize monoculture supported pre-Columbian urbanism in southwestern Amazonia
Source: Nature. 2025 Jan 29;639(8053):119–23. doi: 10.1038/s41586-024-08473-y (PMC11882439; doi:10.1038/s41586-024-08473-y)
Supplement: Supplementary file 2 — Reporting Summary [file 41586_2024_8473_MOESM2_ESM.pdf]

nature portfolio

Corresponding author(s): Umberto Lombardo

Last updated by author(s): Oct 21, 2024

Reporting Summary

Nature Portfolio wishes to improve the reproducibility of the work that we publish. This form provides structure for consistency and transparency in reporting. For further information on Nature Portfolio policies, see our Editorial Policies and the Editorial Policy Checklist.

Statistics

For all statistical analyses, confirm that the following items are present in the figure legend, table legend, main text, or Methods section.

n/a	Confirmed	
☐	☒	The exact sample size (*n*) for each experimental group/condition, given as a discrete number and unit of measurement
☐	☒	A statement on whether measurements were taken from distinct samples or whether the same sample was measured repeatedly
☒	☐	The statistical test(s) used AND whether they are one- or two-sided<br>*Only common tests should be described solely by name; describe more complex techniques in the Methods section.*
☒	☐	A description of all covariates tested
☒	☐	A description of any assumptions or corrections, such as tests of normality and adjustment for multiple comparisons
☒	☐	A full description of the statistical parameters including central tendency (e.g. means) or other basic estimates (e.g. regression coefficient) AND variation (e.g. standard deviation) or associated estimates of uncertainty (e.g. confidence intervals)
☒	☐	For null hypothesis testing, the test statistic (e.g. *F*, *t*, *r*) with confidence intervals, effect sizes, degrees of freedom and *P* value noted<br>*Give P values as exact values whenever suitable.*
☐	☒	For Bayesian analysis, information on the choice of priors and Markov chain Monte Carlo settings
☒	☐	For hierarchical and complex designs, identification of the appropriate level for tests and full reporting of outcomes
☒	☐	Estimates of effect sizes (e.g. Cohen's *d*, Pearson's *r*), indicating how they were calculated

Our web collection on statistics for biologists contains articles on many of the points above.

Software and code

Policy information about availability of computer code

Data collection	DJI propetary software (Terramatch version 023.014 and DJITerra version 3.8.0) has been used for the generation of the LIDAR DEM. C2 Version 1.8 has been used to produce phytolith graphs. ArcGis Pro version version 3.3.2 has been used to produce all the mpas.
Data analysis	Radiocarbon ages have been calibrated and modelled using OxCal 4.4.4. The code is available in Supplementary Information

For manuscripts utilizing custom algorithms or software that are central to the research but not yet described in published literature, software must be made available to editors and reviewers. We strongly encourage code deposition in a community repository (e.g. GitHub). See the Nature Portfolio guidelines for submitting code & software for further information.

Data

Policy information about availability of data

All manuscripts must include a data availability statement. This statement should provide the following information, where applicable:

- Accession codes, unique identifiers, or web links for publicly available datasets
- A description of any restrictions on data availability
- For clinical datasets or third party data, please ensure that the statement adheres to our policy

All the phytolith data supporting the findings of this study are available as supplementary files. Phytoliths have been identified using the sources referenced in methods. Pollen has been identified using the Neotropical pollen database (https://research.fit.edu/paleolab/pollen-database/) and the sources referenced in Methods.

Research involving human participants, their data, or biological material

Policy information about studies with human participants or human data. See also policy information about sex, gender (identity/presentation), and sexual orientation and race, ethnicity and racism.

Reporting on sex and gender	N/A
Reporting on race, ethnicity, or other socially relevant groupings	N/A
Population characteristics	N/A
Recruitment	N/A
Ethics oversight	N/A

Note that full information on the approval of the study protocol must also be provided in the manuscript.

Field-specific reporting

Please select the one below that is the best fit for your research. If you are not sure, read the appropriate sections before making your selection.

☐ Life sciences ☐ Behavioural & social sciences ☒ Ecological, evolutionary & environmental sciences

For a reference copy of the document with all sections, see nature.com/documents/nr-reporting-summary-flat.pdf

Ecological, evolutionary & environmental sciences study design

All studies must disclose on these points even when the disclosure is negative.

Study description	Data are quantitative. The study includes mapping of landscape elements based on visual analysis of remote sensing data and LIDAR; soil and subsoil sampling of forest and savannah; 14C of the samples from a farm pond; phytolith extraction and visual (microscope) analysis (counting of individual phytoliths) of samples gathered from forest and savannah (canals, fields and farm ponds); pollen extraction and visual (microscope) analysis (counting of individual pollen grains) of samples gathered from a farm pond.
Research sample	Stratigraphic profiles have been investigated both using a motor corer and excavations. Sediments sampled for phytolith and pollen analysis have been taken from profiles exposed during excavations. Samples are reppresentative of the local environments where they have been taken.
Sampling strategy	Samples have been taken from stratigraphic profiles at different depths. Some profiles were sampled with a 5 cm resolution (i.e. pond 690), others at 10 cm resolution. The depth of each sample is indicated in the Y axe of each phytolith or pollen graph.
Data collection	Samples have been collected in the field and air-dried in Bolivia before being shipped. Charcoal fragments and vegetal remains for 14C have been collected in situ, enveloped in aluminium foil and stored in plastic bags. Field observations have been wrote down on a notebook. The researchers were aware of the study hypothesis at the time of sampling.
Timing and spatial scale	Sampling has been done in 2021. Lidar has been done in 2023. Samples have been taken in the Beni department, Bolivia.
Data exclusions	No data was excluded
Reproducibility	The experiments consisted in counting a standard number (200) of diagnostic phytoliths and pollen grains. This number is considered sufficient to be representative of the sample, therefore it is not standard practice to repeat the counting.
Randomization	Sampling was not completely random. We identified several potential locations to sample on satellite imagery, we choose those to excavate based on their accessibility and ownership of the land. However, none of these criteria affect the representativeness of our sample.
Blinding	Sampling was not blind because we sampled soil and subsoil, so we knew the origin of each sample. In the lab samples where coded with numbers. Sample extraction and phytolith counting was blind because the origin of the sample was unknown during these steps. Pollen counting was not blind, because we analyzed pollen from only 1 profile, and this was known.

Did the study involve field work? ☒ Yes ☐ No

Field work, collection and transport

Field conditions	Sampling has always being performed during the dry season, between August and October

Location	Fieldwork took place in the Beni department, Bolivia. The area surveyed is enclosed in a square area: up right corner lat -14° 50" Lon -64° 10"; down left corner Lat -15°; Lon -64° 45". Average elevation 180 ma s l. All sampling was performed on land.
Access & import/export	Field sites have been accessed with the permission of the land owner. Authorizations for export of samples have been obtained by the Bolivian Ministry of Medioambiente y Agua (MMAYANMABCCGDF/DGBAP/MEGN°0342/2021) Autorization for flying the drone with the LIDAR has been obtained by DSO - OPERACIONES, Dirección General de Aeronáutica Civil de Bolivia (1ZNBL13)
Disturbance	All the savannah coring and excavation have been performed on land used for pasture with almost complete absence of wild fauna. The test pits in the forest where very small and we didn't produce any noise during the excavation or the sampling. The excavation pits were refilled with the excavated sediments in order to restore the aspect of the sites previous to the excavation.

Reporting for specific materials, systems and methods

We require information from authors about some types of materials, experimental systems and methods used in many studies. Here, indicate whether each material, system or method listed is relevant to your study. If you are not sure if a list item applies to your research, read the appropriate section before selecting a response.

Materials & experimental systems

n/a	Involved in the study
☒	☐ Antibodies
☒	☐ Eukaryotic cell lines
☒	☐ Palaeontology and archaeology
☒	☐ Animals and other organisms
☒	☐ Clinical data
☒	☐ Dual use research of concern
☒	☐ Plants

Methods

n/a	Involved in the study
☒	☐ ChIP-seq
☒	☐ Flow cytometry
☒	☐ MRI-based neuroimaging

Plants

Seed stocks	*Report on the source of all seed stocks or other plant material used. If applicable, state the seed stock centre and catalogue number. If plant specimens were collected from the field, describe the collection location, date and sampling procedures.*
Novel plant genotypes	*Describe the methods by which all novel plant genotypes were produced. This includes those generated by transgenic approaches, gene editing, chemical/radiation-based mutagenesis and hybridization. For transgenic lines, describe the transformation method, the number of independent lines analyzed and the generation upon which experiments were performed. For gene-edited lines, describe the editor used, the endogenous sequence targeted for editing, the targeting guide RNA sequence (if applicable) and how the editor was applied.*
Authentication	*Describe any authentication procedures for each seed stock used or novel genotype generated. Describe any experiments used to assess the effect of a mutation and, where applicable, how potential secondary effects (e.g. second site T-DNA insertions, mosiacism, off-target gene editing) were examined.*
